# Insights into the Mechanism of Human Deiodinase 1

**DOI:** 10.3390/ijms23105361

**Published:** 2022-05-11

**Authors:** Alfonso Rodriguez-Ruiz, Doreen Braun, Simon Pflug, Alexander Brol, Marc Sylvester, Clemens Steegborn, Ulrich Schweizer

**Affiliations:** 1 Institut für Biochemie und Molekularbiologie, Universitätsklinikum Bonn, Rheinische Friedrich-Wilhelms-Universität Bonn, 53115 Bonn, Germany; alfil_311086@hotmail.com (A.R.-R.); dbraun@uni-bonn.de (D.B.); s6sipflu@uni-bonn.de (S.P.); s4albrol@uni-bonn.de (A.B.); 2 Core Facility Mass Spectrometry, Universitätsklinikum Bonn, Rheinische Friedrich-Wilhelms-Universität Bonn, 53115 Bonn, Germany; msylvest@uni-bonn.de; 3 Lehrstuhl Biochemie, Universität Bayreuth, 95447 Bayreuth, Germany; clemens.steegborn@uni-bayreuth.de

**Keywords:** selenoprotein, selenocysteine, thyroid hormone, kinetic analysis, mass spectrometry

## Abstract

The three isoenzymes of iodothyronine deiodinases (DIO1-3) are membrane-anchored homo-dimeric selenoproteins which share the thioredoxin-fold structure. Several questions regarding their catalytic mechanisms still remain open. Here, we addressed the roles of several cysteines which are conserved among deiodinase isoenzymes and asked whether they may contribute to dimerization and reduction of the oxidized enzyme with physiological reductants. We also asked whether amino acids previously identified in DIO3 play the same role in DIO1. Human DIO1 and 2 were recombinantly expressed in insect cells with selenocysteine replaced with cysteine (DIO1^U126C^) or in COS7 cells as selenoprotein. Enzyme activities were studied by radioactive deiodination assays with physiological reducing agents and recombinant proteins were characterized by mass spectrometry. Mutation of Cys124 in DIO1 prevented reduction by glutathione, while 20 mM dithiothreitol still regenerated the enzyme. Protein thiol reductants, thioredoxin and glutaredoxin, did not reduce DIO1^U126C^. Mass spectrometry demonstrated the formation of an intracellular disulfide between the side-chains of Cys124 and Cys(Sec)126. We conclude that the proximal Cys124 forms a selenenyl-sulfide with the catalytic Sec126 during catalysis, which is the substrate of the physiological reductant glutathione. Mutagenesis studies support the idea of a proton-relay pathway from solvent to substrate that is shared between DIO1 and DIO3.

## 1. Introduction

Thyroid hormones (TH) profoundly regulate metabolism, growth, cell differentiation, and development [1,2,3]. Thyroxine (3,3′,5,5′-tetraiodothyronine, T_4_) is the main product of the thyroid gland, while 3,3′,5-triiodothyronine (T_3_) is the principal receptor-binding molecule interacting with the nuclear T_3_-receptors [4]. Similar to other ligands of nuclear receptors, access of ligand to receptor is regulated on at least four levels: (i) distribution in the blood stream through transfer proteins, (ii) cellular uptake across the cell membrane, (iii) local activation of a precursor to form the active ligand, and (iv) inactivation of ligands. The latter two processes are mediated by iodothyronine deiodinase isoenzymes (DIO1-3). For example, 5′-deiodination of T_4_ catalyzed by DIO1 or DIO2 activates the prohormone T_4_ to the active T_3_. 5-deiodination of T_4_ and T_3_ by DIO3 leads to reverse T_3_ (3,3′,5′-triiodothyronine, rT_3_) and 3,3′-T_2_, respectively, two iodothyronines not capable of activating T_3_ receptors [4]. 

Iodothyronine deiodinases are specific for metazoans and contain in mammals the rare amino acid selenocysteine (Sec), which is considered a superior catalyst compared to cysteine (Cys), although Cys-containing deiodinases exist in invertebrates and replacement of Cys for Sec does not abrogate catalytic activity of mutated deiodinases [5,6,7]. From a structural point of view, deiodinases share a thioredoxin fold similar to peroxiredoxins [8]. It is now clear that the Sec in the active center forms a halogen bond with the substrate iodine and abstracts an iodonium (I^+^) forming a selenenyl-iodide intermediate, while the substrate takes up a proton [9]. How the selenenyl-iodide is reduced is not entirely clear. One possibility is the rapid exchange of iodide (I^−^) with hydroxyl (HO^−^) in the aqueous environment, forming a selenenic acid intermediate as known from glutathione peroxidases. This intermediate or the selenenyl-iodide is then reduced by thiols. In vitro, dithiothreitol (DTT) is generally used as a potent reducing co-factor of deiodinases. Physiological reductants including glutathione (GSH), thioredoxin (TXN), and peroxiredoxin 3 (PRDX3) have been proposed for deiodinases [10,11,12,13], but the exact mechanism of the re-reduction of the individual deiodinase isoenzymes has not been clarified [14]. A related question is how the proton is transferred to the substrate. We have proposed that a hydrogen-bonded network of amino acid side chains involving several conserved positions in all three deiodinase isoenzymes relays a proton from solvent to the substrate [8]. Based on molecular dynamics calculations, Bayse et al. have recently proposed that these amino acids are involved in forming the substrate-binding pocket [15]. Another open question relates to the known dimerization of deiodinases. It is clear that substrate binding requires formation of the dimer [16], and it is known that the transmembrane anchor of deiodinases contributes to the formation of the dimer. However, it is not clear how the deiodinase dimer is stabilized, where the dimerization interface lies, and whether the linker between transmembrane helix and catalytic domain contributes to dimerization and/or substrate binding. Finally, structural information is missing to explain the different substrate specificities and regioselectivities of the three isoenzymes [14].

Here, we addressed several of the above questions and investigated the roles of several cysteines, which are conserved among vertebrate species, in human DIO1 with regard to dimerization and catalysis. We demonstrate for the first time directly a disulfide between the catalytic Sec(Cys)126 and the proximal Cys124 in DIO1. We further show that Cys124 is irrelevant if DTT is used as a reducing co-factor, but reduction by GSH is sensitive to availability of Cys124. Thus, we conclude that the physiological cofactor GSH likely attacks the selenenyl-sulfide in DIO1 that results from intramolecular reduction of the primary selenenyl-iodide (or selenenic acid) intermediate. We then investigated in DIO1 by mutagenesis the amino acids previously proposed to participate in the proton relay in DIO3. Our analysis supports a similar mechanism of proton transfer in DIO1 without ruling out a role of these amino acids in formation of the substrate-binding pocket. 

## 2. Results

### 2.1. Conserved Amino Acids among Deiodinase Isoenzymes

**Figure 1:**
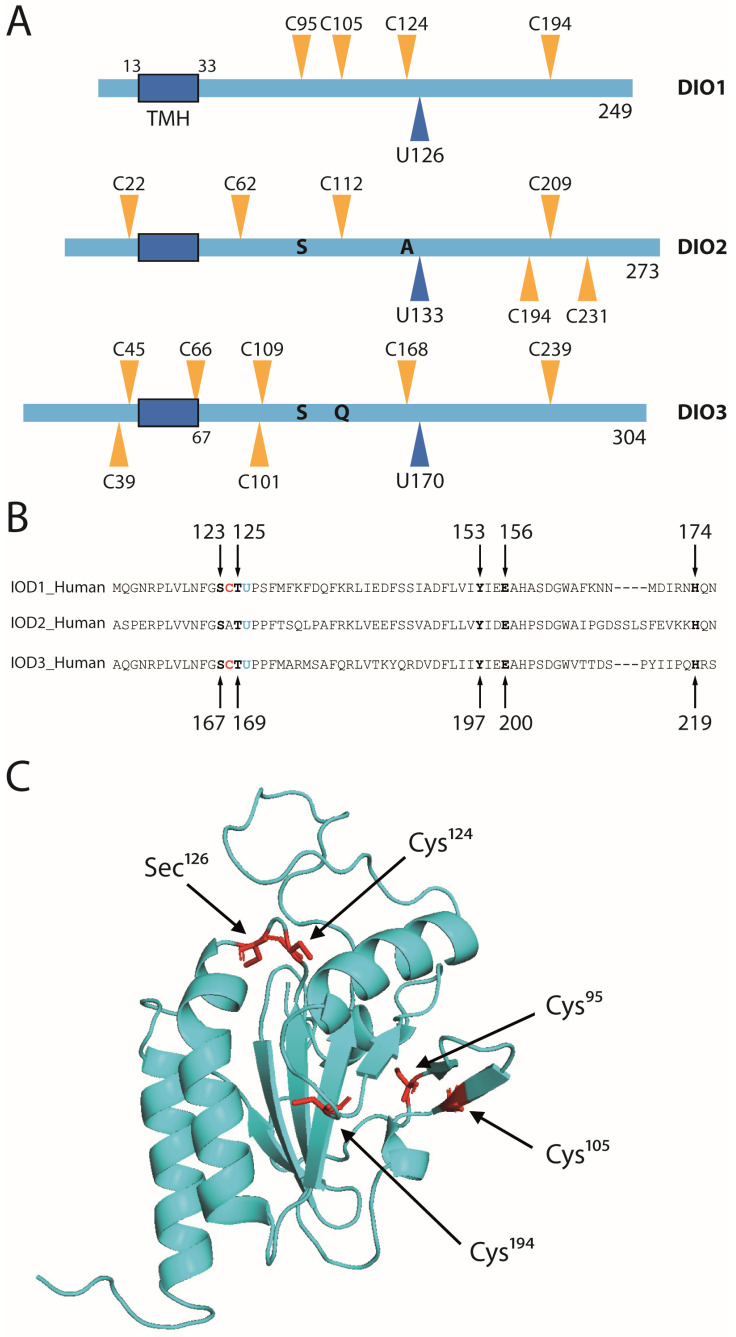
Conserved amino acid positions in deiodinases. (**A**). Cysteines in human DIO1-3. TMH transmembrane helix. (**B**). Primary sequence surrounding the catalytic Sec (U) and Cys (C) in DIO1-3. (**C**). Spatial orientation of conserved cysteines. Homology model of human DIO1 based on mDIO3^cat^ (PDB 4TR4).

**Figure A1:**
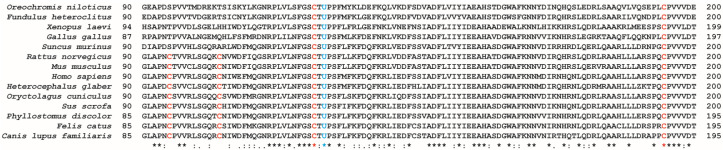
Multiple alignment of vertebrate DIO1 peptide sequences indicating conserved cysteines (red) and selenocysteine (blue). The alignment was performed with CLUSTAL W. * invariant amino acid. SwissProt identifiers correspond to the following species: *Oreochromis niloticus* (Nile tilapia), *Fundulus heteroclitus* (Atlantic killifish), *Xenopus laevis* (African clawed frog), *Gallus gallus* (domestic chicken), *Suncus murinus* (Asian house shrew), *Rattus norvegicus* (brown rat), *Mus musculus* (house mouse), *Homo sapiens* (human being), *Heterocephalus glaber* (naked mole-rat) (protein inferred from homology), *Oryctolagus cuniculus* (European rabbit), *Sus scrofa* (domestic pig), *Phyllostomus discolor* (pale spear-nosed bat) (protein inferred from homology), *Felis catus* (domestic cat), and *Canis lupus familiaris* (domestic dog).

The schematic shows the positions of cysteines and the catalytic selenocysteine in human DIO1-3 (Figure 1A). With respect to the catalytic selenocysteine, one distal cysteine (Cys194 in DIO1) is conserved among all isoenzymes, 69–76 amino acid positions C-terminal from the selenocysteine. The proximal cysteine (Cys124 in DIO1) is conserved among DIO1 and DIO3, but is replaced with alanine in DIO2 (Figure 1B). Both cysteines at positions 95 and 105 are conserved in mammalian DIO1 sequences, but are absent in birds and amphibians (Figure A1). The immediate context of the catalytic selenocysteine is highly conserved among DIO1-3 (Figure 1B). Based on the hydrogen-bonding network observed in the crystal structure of the catalytic domain of murine DIO3, the conserved Thr125, Glu156, and His174 (in DIO1 numbering) have been proposed by us as participating in relaying a proton from the solvent to the substrate [8]. The spatial relationship of the amino acids discussed is evident in the homology model of DIO1 based on murine DIO3 (Figure 1C).

### 2.2. Is There an Intermolecular Disulfide in the Mammalian DIO1 Homodimer?

**Figure 2:**
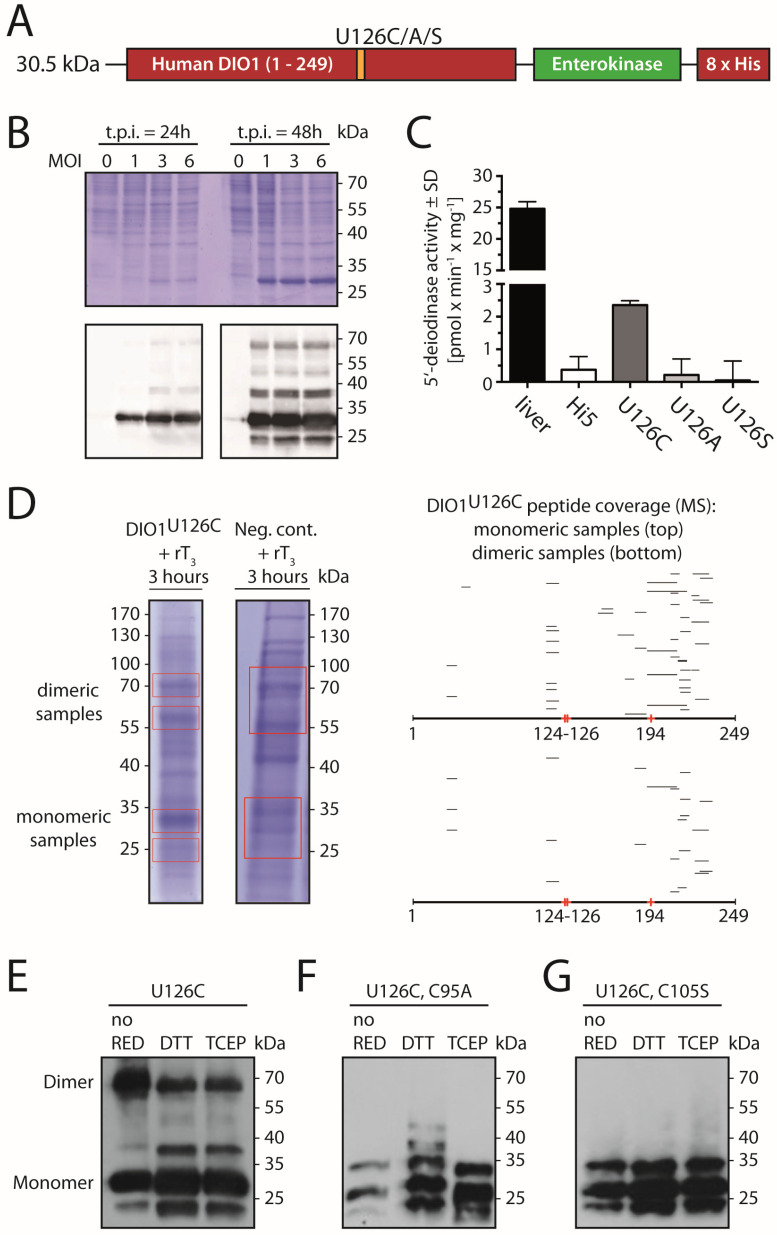
Biochemical characterization of recombinant human DIO1^U126C^ expressed in insect cells. (**A**). Construct. A C-terminal His-tag was added to the human DIO1 sequence separated by an enterokinase cleavage site. (**B**). Electrophoretic mobility of DIO1^U126C^ expressed in Hi5 cells 24 h and 48 h post-infection (t.p.i.) with baculovirus. MOI, multiplicity of infection. Upper panel Coomassie-stained gel, lower panel immunodetection with anti-His-tag antibody. (**C**). Catalytic activity of recombinant DIO1^U126C^ compared to negative controls (DIO1^U126A^, DIO1^U126S^, and non-infected Hi5 cells). Mouse liver homogenate served as positive control. SD standard deviation. (**D**). Detection of DIO1^U126C^-derived peptides by mass spectrometry. Regions encompassing “monomeric” and “dimeric” bands were cut from Coomassie-stained gels loaded with protein from DIO1^U126C^-expressing cells and non-infected controls after incubation with rT3 for 3 h. Right panel, peptide coverage of DIO1 found in tryptic digests of gel slices. (**E**–**G**). Western blotting against His-tag following non-reducing SDS-PAGE of recombinant DIO1 pretreated with or without reductants (DTT or TCEP).

We wanted to directly investigate whether conserved cysteines in DIO1 participate in redox reactions to form disulfides. However, selenoproteins are notorious for their low expression levels and deiodinases are membrane proteins, further complicating purification and biochemical analysis of native protein. To access significant amounts of DIO1 protein, DIO1 has been previously expressed in *Saccharomyces cerevisiae* at high yield, but was not catalytically active [17]. We, therefore, decided to express human DIO1 recombinantly in High5 insect cells using a baculoviral transduction system. As these insect cells do not harbor the machinery for selenoprotein biosynthesis, we decided to express DIO1 with Sec replaced by Cys (DIO1^U126C^). In order to facilitate the immunodetection of the recombinant enzyme, we added a C-terminal 8xHis-tag preceded by an enterokinase cleavage site (Figure 2A).

Twenty-four hours after transfection, DIO1 expression was readily detected by Western blotting (Figure 2B). At a multiplicity of infection (MOI) of 3 and 48 h post transduction, expression of DIO1^U126C^ was easily seen in the Coomassie-stained SDS-PAGE gel. DIO1^U126C^ in membrane-enriched protein fraction from Hi5 cells was catalytically active in the common radioactive 5′-deiodinase assay using ^125^I-rT3 and DTT as reductant. Non-transduced Hi5 cells and cells expressing catalytically inactive DIO1^U126A^ or DIO1^U126S^ did not show any background deiodinase activity. The inclusion of mouse liver served as a positive control for the enzymatic assay (Figure 2C). A specific activity of about 10% of the wild-type enzyme is expected for DIO1 with the catalytic Sec replaced by Cys [5].

In the Coomassie-stained gel as well as in the immunoblot against the His-tag, we found a band at a mobility corresponding to 60 kDa of which we wondered whether it might represent a DIO1 dimer. We therefore cut these bands from the gel and subjected them to mass-spectrometric analysis (Figure 2D). Both the major 30 kDa and the 60 kDa “dimer” band contained DIO1 peptides, suggesting that the material migrating in the 60 kDa band likely represents a DIO1 homodimer. Since this dimer appeared stable even after incubation with Laemmli buffer containing 5% β-mercaptoethanol, we wondered whether it was stabilized by a disulfide bond (Figure 2B).

We speculated that the two conserved cysteines in mammalian DIO1, C95 and C105, might be involved in formation of a stable homodimer of DIO1 (Figure A1). Thus, we expressed DIO1^U126C^ recombinant protein with Cys95 and Cys105 replaced with Ala (C95A) and Ser (C105S), respectively. DIO1^U126C^, DIO1^U126C/C95A^, and DIO1^U126C/C105S^ proteins were analyzed by non-reducing SDS-PAGE followed by Western blotting after preincubation or not with the reductants, 20 mM DTT and 20 mM TCEP (tris(2-carboxyethyl)phosphine), respectively (Figure 2E–G). While the dimeric band was readily observed in DIO1^U126C^ without reduction, the band intensity was decreased after incubation with either reductant (Figure 2E). In contrast, DIO1^U126C/C95A^ protein did not show any dimer band in Western blots (Figure 2F). Likewise, DIO1^U126C/C105S^ protein did not show a dimer band (Figure 2G). Based on these results, we thought it possible that DIO1^U126C^ protein forms a disulfide-stabilized dimer. 

**Figure A2:**
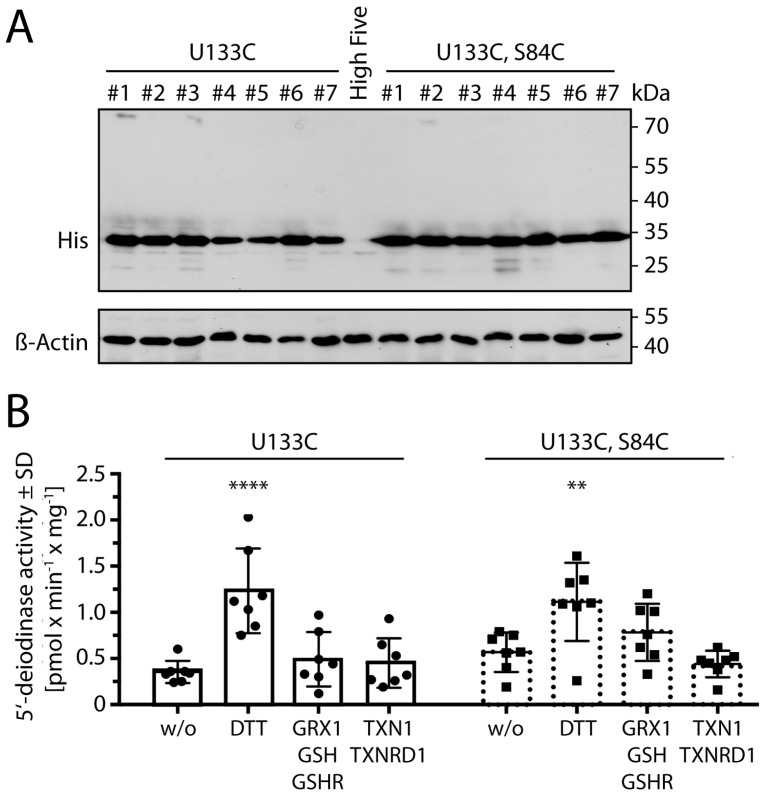
Recombinant expression of human DIO2 in insect cells. (**A**). Western blot against the C-terminal His-tag of DIO2^U133C^ and DIO2^U133C/S84C^. Shown are independent transductions with viral particles. High Five denotes non-transduced cells which served as a negative control. Western blot against β-actin was used to show similar protein content. (**B**). 5′-deiodinase activity of insect cells transduced with human DIO2^U133C^-expressing constructs in relation to the reducing co-factors used. DIO2^U133C^ exhibits activity in the presence of the artificial reductant DTT (20 mM), while physiological regenerating reducing systems with 1 mM GSH, 0.05 µM GSHR, and 200 µM NADPH; or 3 µM TXN1 and 0.1 µM TXNRD1; and 200 µM NADPH did not support activity beyond the first turnover that occurs even in the absence of reductant (“w/o”). The mutation S84C does not increase activity. ** *p* < 0.01, **** *p* < 0.0001, Student’s *t* test compared to enzyme without (w/o) reductant. Symbols denote means of individual experiments (#1–7) performed in triplicates. SD: standard deviation.

Motivated by the idea that stabilization of the DIO1 dimer might contribute to the much higher catalytic activity of DIO1 compared to DIO2, we wondered whether this idea could be tested by creating a disulfide-stabilized DIO2. Cys105 of DIO1 corresponds to Cys112 in DIO2, while DIO2 carries a Ser84 at the position corresponding to Cys95 in DIO1 (Figure 1A). We therefore recombinantly expressed human DIO2^U133C^ and the corresponding DIO2^U133C/S84C^ in insect cells (Figure A2). Western blot analysis detected only monomeric DIO2^U133C^ with or without the S84C exchange. Further, while the DIO2^U133C^ enzyme showed considerable activity in the presence of DTT, this activity did not increase with the S84C mutation. These results from DIO2 do not refute the possibility of a disulfide in dimeric DIO1, but also do not support it. However, both cysteines are expected to be on the surface of DIO1, close to the small β-sheet β1 (Figure 1C), but this location would imply the disulfide bridge is formed in the cytosol in the physiological system, which is known to be rare.

### 2.3. Conserved Cysteines in the Mechanism of Reduction of DIO1

**Figure 3:**
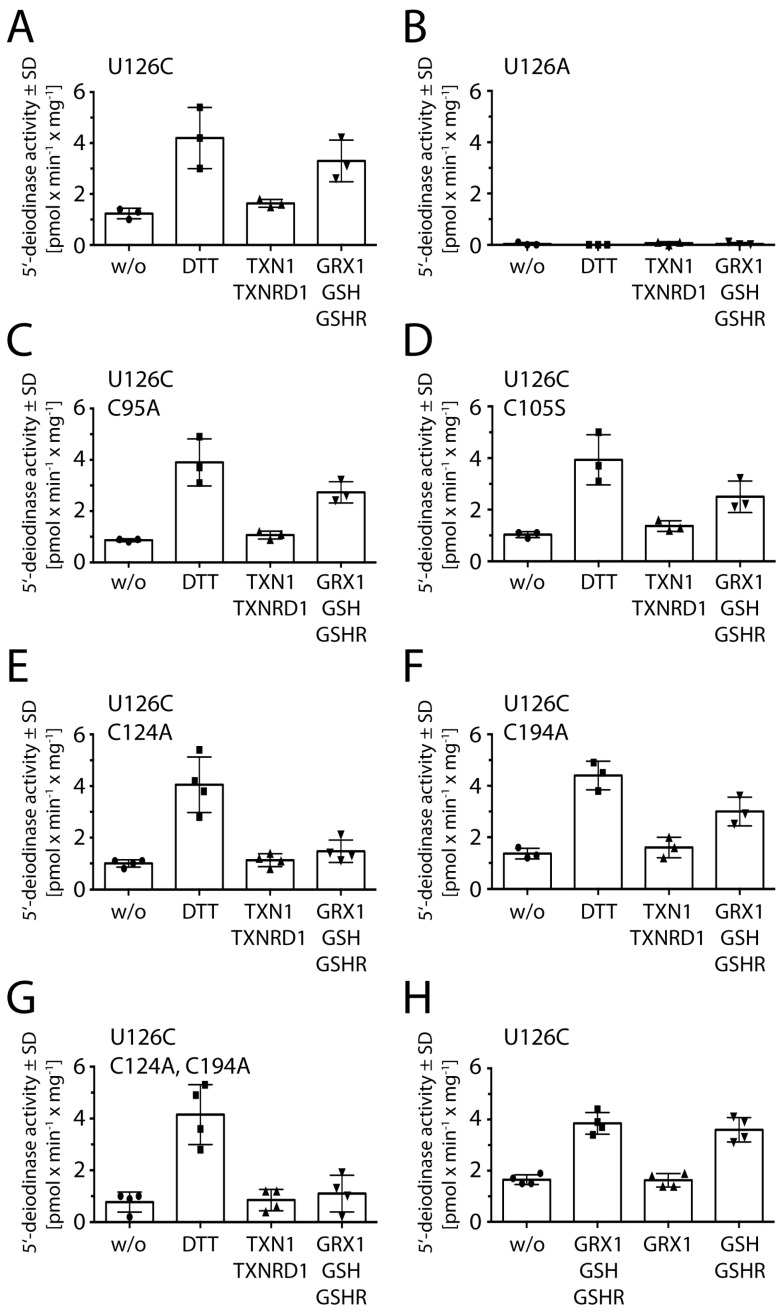
5′-deiodinase activity of recombinant purified DIO1^U126C^ variants using different reducing systems. (**A**). DIO1^U126C^, (**B**). DIO1^U126A^ served as a negative control, (**C**). DIO1^U126C/C95A^, (**D**). DIO1^U126C/C105S^, (**E**). DIO1^U126C/C124A^, (**F**). DIO1^U126C/C194A^, (**G**). DIO1^U126C/C124A/C194A^. (**H**). DIO1^U126C^ is not active with GRX alone, but requires GSH. w/o: without reductant, 20 mM DTT; 3 µM TXN1 and 0.1 µM TXNRD1; and 1 mM GSH, 1 µM GRX1, and 0.05 µM GSHR; and 200 µM NADPH. The experiment was performed with protein from 3 to 4 different transductions of Hi5 cells. SD: standard deviation.

Our previous work on murine DIO3 focused on the roles of Cys168 and Cys239 in reduction of the enzyme [8]. In order to see whether reduction of DIO1 is similar to reduction of DIO3, we mutated the corresponding cysteines at positions 124 and 194 in DIO1^U126C^ (Figure 1A). We expressed the enzyme in insect cells, extracted the membrane fractions comprising the recombinant enzyme, and performed activity assays using three different reducing systems, i.e. DTT, a thioredoxin-regenerating system (thioredoxin 1, TXN1; thioredoxin reductase 1, TXNRD1; and NADPH), and a glutathione-regenerating system (glutathione, GSH; glutaredoxin 1, GRX1; glutathione reductase, GSHR; and NADPH) (Figure 3).

We found that a regenerating TXN system containing TXNRD1 and NADPH was not able to reduce DIO1^U126C^, while the regenerating system with 1 mM GSH and GRX1 was able to support the enzymatic reaction (Figure 3A). A DIO1^U126A^ mutant served as negative control (Figure 3B). Mutation of either Cys95 or Cys105 did not reduce the activity of recombinant DIO1^U126C^ with DTT or the GSH-reducing system (Figure 3C,D). This finding again argues against a functional role of the potential intermolecular disulfides at these positions. 

Mutation of Cys124 did not affect reduction by DTT, but reduction by the regenerating GSH system (Figure 3E). Mutation of Cys194 did not change reduction by DTT and did not significantly reduce reduction by the GSH system (Figure 3F). A double mutant of Cys194 and Cys124 did not affect reduction by DTT, but abolished reduction by the GSH system (Figure 3G)—as did C124A alone. This result shows that Cys124, but not Cys194, is required for interaction with GSH. 

**Figure A3:**
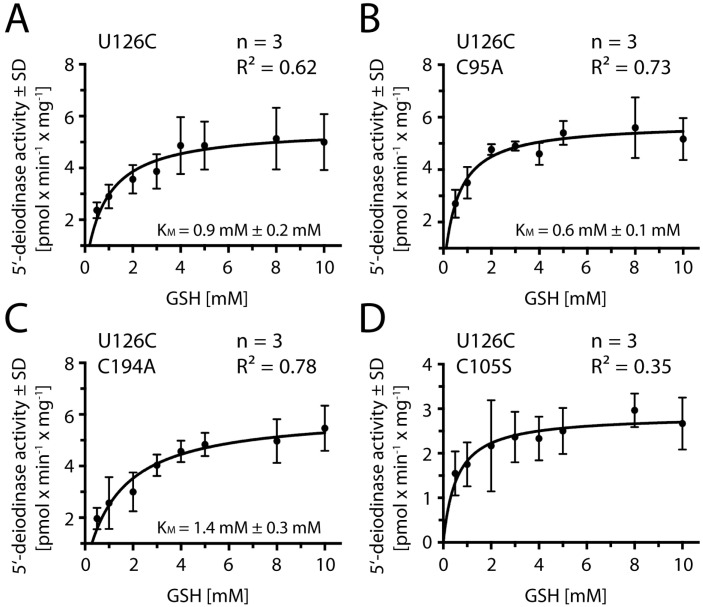
Kinetic analysis of human DIO1^U126C^ in insect cells. Michaelis constants (K_M_) for GSH do not depend on cysteines 95, 105, or 194. (**A**). DIO1^U126C^. (**B**). DIO1^U126C/C95A^. (**C**). DIO1^U126C/C194A^. (**D**). DIO1^U126C/C105S^. Experiment performed in triplicates. Correlation coefficients of fits (R^2^) and K_M_ ± SD are indicated. Curve fitting was performed with GraphPad Prism. SD: standard deviation.

Then, we wondered whether the protein thiol GRX was needed at all in the regenerating reducing system and performed the activity assay with DIO1^U126C^ in the presence of the complete system, only GRX, and only GSH and GSHR (Figure 3H). We found that 1 mM GSH is sufficient to reduce DIO1^U126C^ in vitro, if GSH is reduced by GSHR and NADPH. Additional kinetic analyses determining the K_M_ value for GSH of the various DIO1 variants yielded a K_M_ for GSH of the DIO1^U126C^ protein of 0.9 ± 0.2 mM (Figure A3). Hence, GSH is likely a physiological reducing agent in vivo of DIO1. 

### 2.4. Analysis of Cysteine Disulfides in Recombinant DIO1^U126C^ by Mass Spectrometry

We wanted to harness the specific advantage of the availability of high amounts of DIO1 from insect cells and attempt the identification of potential inter- and intramolecular disulfides in DIO1^U126C^. We thus incubated DIO1^U126C^ variants with or without rT_3_, blocked thiols with N-Ethylmaleimide (NEM), and performed in-gel digestion with chymotrypsin/trypsin followed by mass spectrometry (see Materials and Methods in Section 4). Our idea was to specifically search for evidence of Cys95-Cys95, Cys95-Cys105, or Cys105-Cys105 intermolecular disulfides in the 60 kDa “dimeric” band of our recombinant enzyme preparation. However, no evidence for such peptides was found.

**Figure 4:**
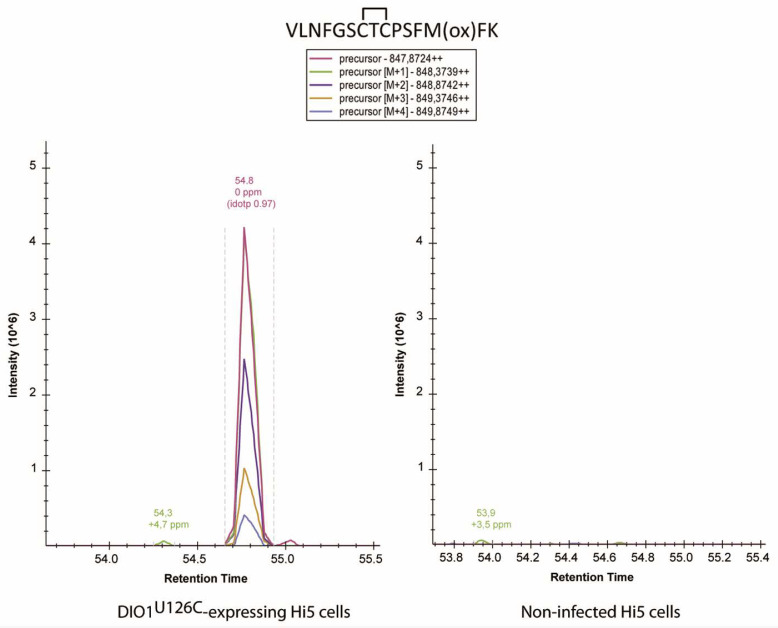
Evidence of an intramolecular disulfide in recombinant DIO1^U126C^. Extracted ion chromatograms of peptide VLNFGSCTCPSFMFK comprising the Cys124-Cys126 disulfide bond in the negative controls (non-transfected Hi5 cells, right panel) and DIO1^U126C^-expressing cells (left panel). The figure was obtained using the Skyline software.

**Figure 5:**
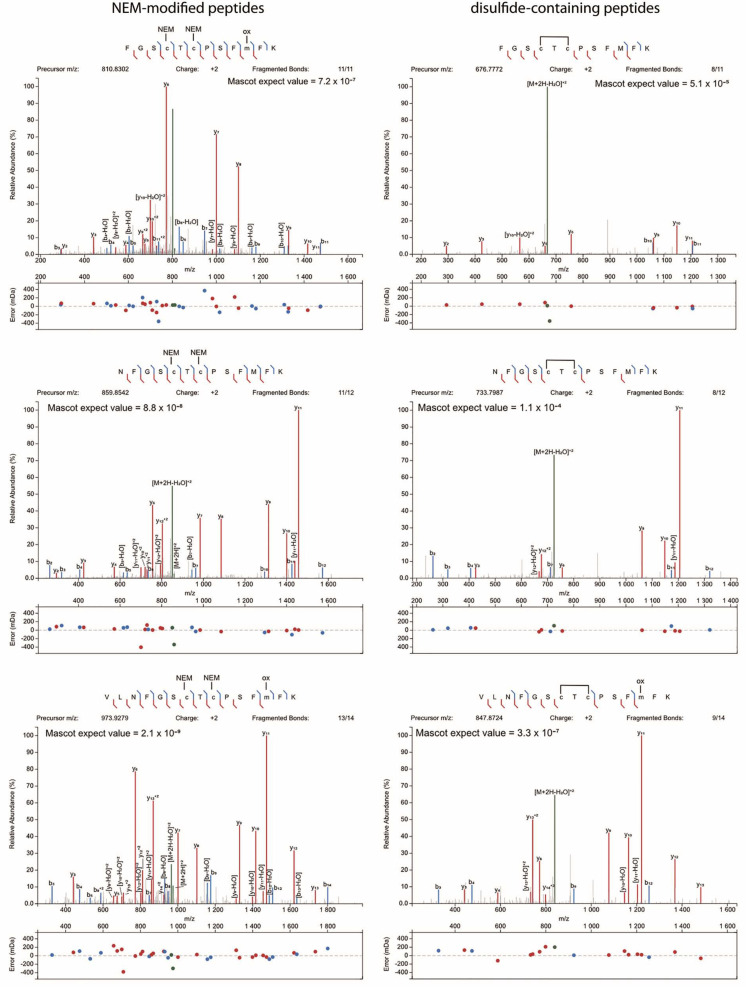
Evidence of an intramolecular disulfide in recombinant DIO1^U126C^. Comparison of MS2 spectra of differently modified peptide ions comprising positions Cys124-Cys126 in DIO1^U126C^ samples incubated with reverse triiodothyronine (rT_3_). Displayed are spectra for peptides with NEM modifications on Cys124 and Cys126 (left panels) and for peptides with intramolecular disulfide bridge on the same amino acid residues (right panels). Annotations on the MS2 spectra were performed employing Peptide Annotator: https://www.interactivepeptidespectralannotator.com/PeptideAnnotator.html, accessed on 1 January 2020.

**Figure A4:**
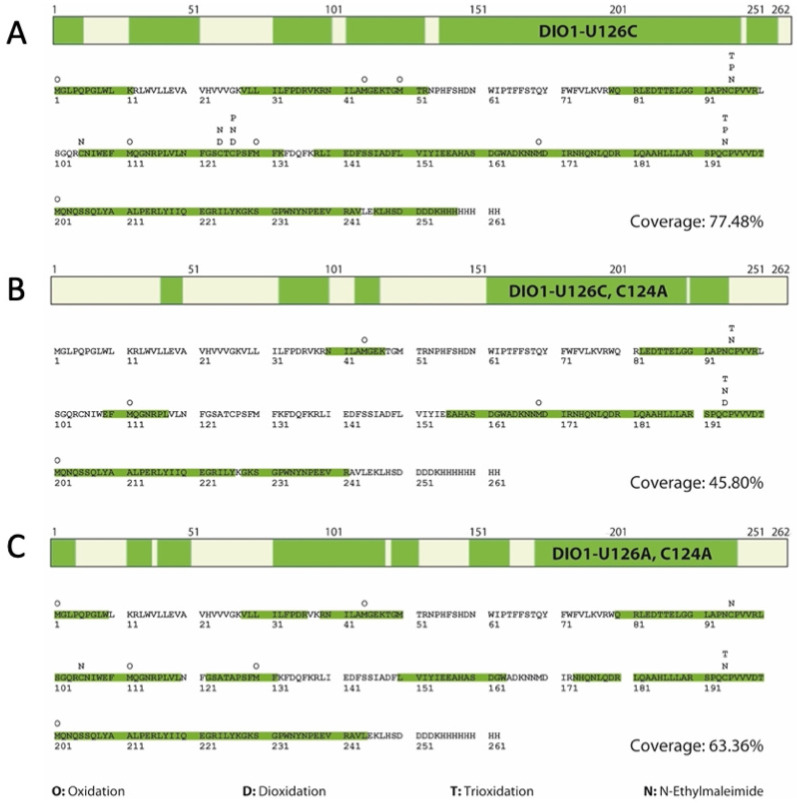
Mass spectrometry of recombinant DIO1^U126C^ variants. Coverage of the peptide sequence for the different mutants recombinantly expressed in Hi5 cells: (**A**): DIO1^U126C^; (**B**). DIO1^U126C/C124A^; (**C**). DIO1^U126A/C124A^. Note the dehydro modifications at positions 124 and 126 in the DIO1^U126C^ mutant as well as the absence of sequence coverage around the catalytic center for the DIO1 ^U126C/C124A^ mutant.

We then searched for intramolecular disulfides, Cys95-Cys105, Cys124-Cys126, Cys126-Cys194, Cys124-Cys194, and other possible combinations in both the “dimeric” and monomeric bands. These analyses were performed with enzyme preparations exposed or not to rT3 as a substrate to induce oxidation of the enzyme. Obviously, reductants were neither added to the rT3 reaction mix nor to the SDS-PAGE. Rather, free thiols were blocked by NEM before electrophoresis. Of all disulfides we have searched for, only one could be clearly demonstrated, i.e. a Cys124-Cys126 intramolecular disulfide in the 30 kDa band (Figure 4 and Figure A4). Accordingly, evidence for this species was absent in DIO1^U126A^ and DIO1^C124A^ variants. More precisely, three peptides with different N-termini containing the intramolecular disulfide were detected along with their NEM-modified forms (Figure 5). While a selenenyl-sulfide/disulfide between the catalytic SeCys/Cys and the proximal Cys has been postulated previously, this is the first report to show direct evidence for the existence of such an intramolecular Cys124-Cys126 disulfide.

### 2.5. Cys124 Is Essential for Reduction with GSH in DIO1 Containing Selenocysteine

**Figure 6:**
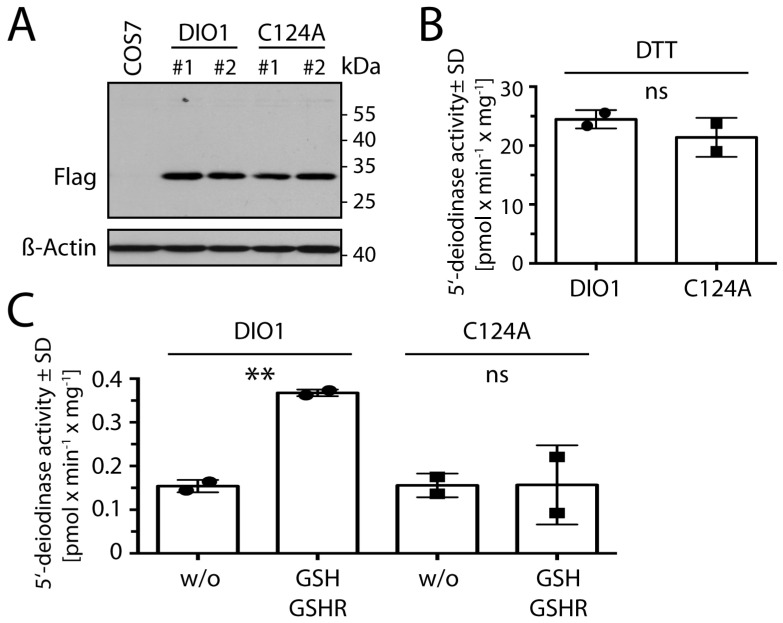
Cys124 is required for reduction by GSH of DIO1 containing Sec. COS7 cells were transfected with human DIO1 constructs containing an N-terminal Flag-tag. (**A**). Expression of DIO1 constructs in COS7 cells detected by Western blot with an anti-Flag antibody. β-Actin served as loading control. (**B**). 5′-deiodinase activity of transfected cells in the presence of 20 mM DTT. (**C**). 5′-deiodinase activity of DIO1 or DIO1^C124A^ in the presence of a regenerating reducing system comprising 1 mM GSH, 0.05 µM GSHR, and 200 µM NADPH; w/o: without reducing co-factor, ** *p* < 0.01, Student’s *t*-test. SD: standard deviation. Two independent transfections (#1 and #2) measured in triplicates; shown are the means of the triplicates; ns not significant.

In order to confirm the relevance of the intramolecular Cys124-Cys126 disulfide in the context of selenocysteine at position 126, we transfected COS7 cells with expression constructs for DIO1 (containing the UGA/Sec codon) with and without the C124A mutation (Figure 6A). Deiodinase assays with ^125^I-rT3 as substrate showed that the presence of Cys124 was not required if 20 mM DTT was used as a reducing agent (Figure 6B). In the presence of the more physiological reducing system comprised of 1 mM GSH, 0.05 µM GSHR, and 200 µM NADPH, the C124A mutant showed no increase of basal activity through addition of thiol cofactor (Figure 6C). This means that the deiodination reaction does not depend on Cys124, but the re-reduction of the enzyme does. We conclude from this experiment that DTT may replace the thiol from Cys124 in vitro, while GSH apparently is not able to directly interact with oxidized Sec/Cys126 in DIO1. We propose that after oxidation of amino acid 126, the proximal Cys124 attacks the oxidized species and forms the disulfide (selenenyl-sulfide) which we have demonstrated here. Only this species is the physiological substrate for reduction by GSH. 

### 2.6. New Evidence for a Proton Relay Pathway from Solvent to Substrate in DIO1

**Figure 7:**
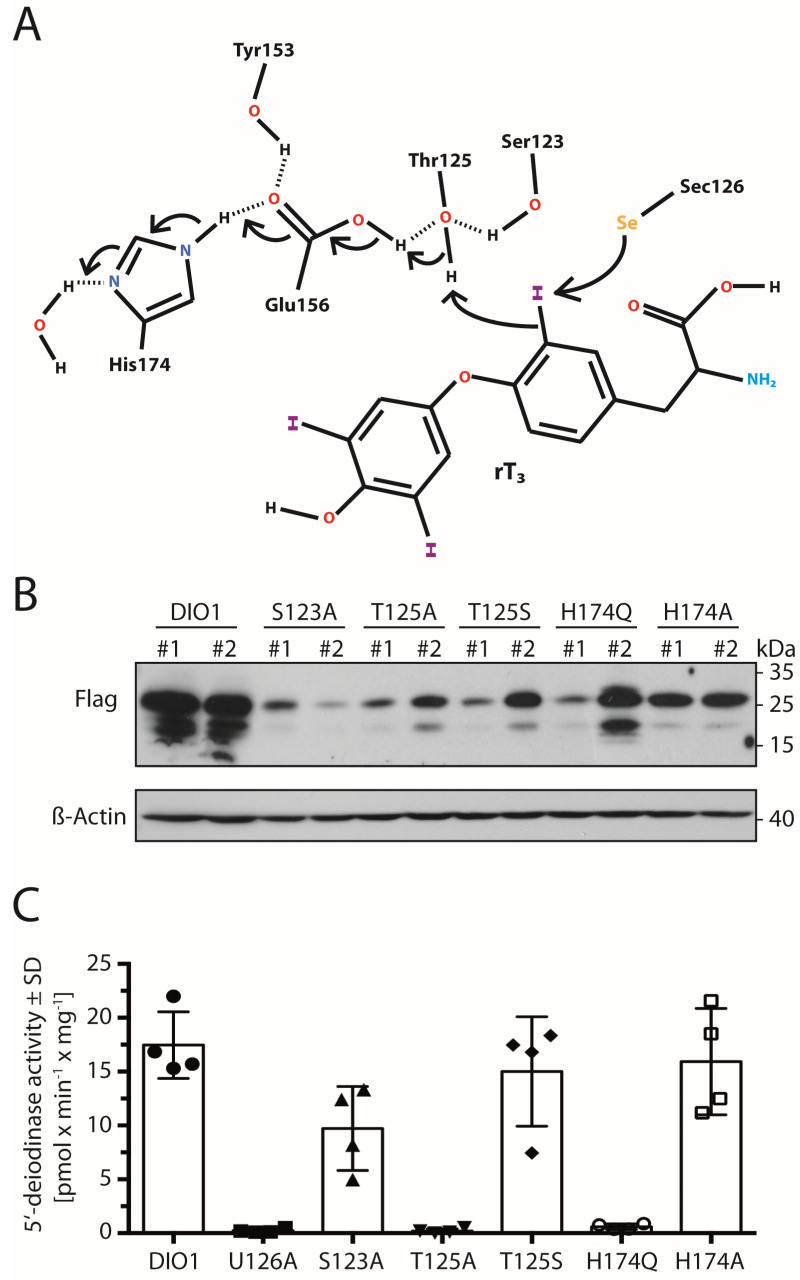
Amino acid residues in DIO1 possibly involved in a proton relay pathway. (**A**). Schematic representation of the conserved hydrogen-bond network. Arrows indicate electron shifts for a potential proton relay. (**B**). Western blot analysis of DIO1 in stable COS7 clones directed against the Flag-tag. (**C**). 5′-deiodinase activities of DIO1 variants expressed in COS7 cells. 20 mM DTT served as reducing co-factor. SD: standard deviation.

**Figure A5:**
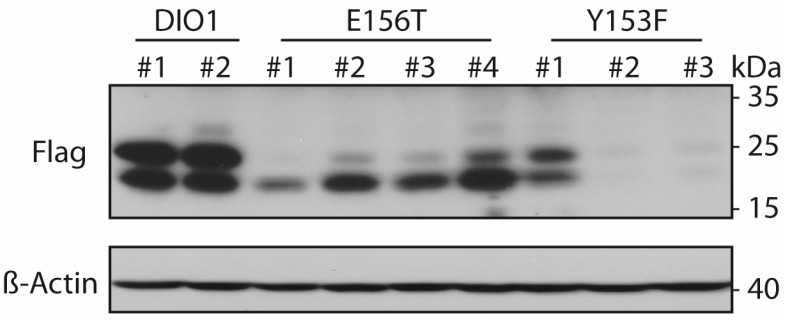
Expression of DIO1 mutants in COS7 cells. Western blot directed against the Flag-tag shows that clones transfected with the E156T and Y153F mutations showed weak expression of full-length DIO1. Deiodinase activities of the E156T and Y153F clones were not significantly higher than non-transfected controls.

According to the alignment between human deiodinase isoenzymes (Figure 1B), all amino acids previously suggested in DIO3 to participate in a hydrogen-bonded proton relay network [8] are conserved in DIO1. We hypothesized that a hydroxyl (Ser123 or Thr125 in DIO1) donates a proton to the substrate after the catalytic (seleno-)cysteine abstracted the iodonium (I^+^) from the iodothyronine. The proton would be replaced by Glu156, which would, in turn, receive a proton from protonated His174, which is exposed to the solvent (Figure 7A). Tyr153 was proposed to stabilize the hydrogen-bonded network. In order to test this hypothesis in DIO1 directly, we mutated these amino acids to structurally related amino acids that, however, do not engage in the same hydrogen-bonding interactions as the original amino acids. Ser123 was mutated to Ala, Thr125 was mutated to Ala and Ser, and His174 was mutated to Ala and Gln. The respective DIO1 mutant proteins were expressed by stable transfection in COS7 cells, and a 5′-deiodinase activity assay was performed with DTT as reductant (Figure 7B). We found that the hydroxyl function of Ser123 is not essential for deiodination, but it seems to be involved in DIO1 activity, since DIO1^S123A^ activity was clearly reduced compared to the wild-type. In contrast, the hydroxyl of Thr125 is essential, as DIO1^T125A^ was entirely inactive, while DIO1^T125S^ retained almost wild-type activity. These findings are consistent with previous results from murine DIO3 [8]. We also mutated Gln156 to Thr and Tyr153 to Phe, but both mutants did not express well in COS7 cells (Figure A5).

Concerning His174, we obtained an interesting finding. An H174Q mutant is expected to engage in similar H-bonding interactions with Glu156 as His, but cannot donate a proton. Accordingly, DIO1^H174Q^ was inactive (Figure 7B). In contrast, DIO1^H174A^ showed normal activity compared to the wild-type enzyme. Based on the DIO3 structure [8], we interpret this finding as Ala174 creating a void on the surface of DIO1 which allows water to access Glu156 and donate the proton directly. The latter result supports the hypothesis of proton transfer from solvent to iodothyronine along the proposed pathway and extends beyond a structural function of the hydrogen-bonded network.

## 3. Discussion

The goal of this study was to better understand the mechanism of deiodinases, in particular DIO1. While iodothyronine substrates and products are well-established, the nature of the reducing co-substrate or co-factor has not been unanimously settled. In particular, because DTT has been established as the most efficient (and cheapest) reducing co-factor of deiodinases, repeated attempts to identify the natural reducing substrate remained fragmented over groups, deiodinase isoforms, species, and time. Moreover, the use of DTT may, in hindsight, have hindered the microscopic understanding of the deiodinase mechanism.

### 3.1. The Mechanism: Re-Reduction of the Oxidized Deiodinase

The current understanding of the oxidative part of the deiodinase mechanism is undisputed: the Sec in the active site is thought to attack the iodine forming a Se-I bond and thereby weaken the carbon-Se bond, formally abstracting an I^+^ from the iodothyronine [4,6,9,18]. The primary oxidized species, i.e., a selenenyl-iodide of a deiodinase has never been observed directly, because it is likely unstable in water. However, a model compound designed to prevent hydrolysis of the Se-I bond has been prepared [19]. Hydrolysis of the selenenyl-iodide would lead to a selenenic acid (Se-OH), which equally has never been demonstrated in a deiodinase. Recently, the studies of the model compound have been extended and it was shown that both the selenenyl iodide and the selenenic acid species readily reacted with thiols [20]. They showed that rapid formation of a selenenyl sulfide is able (and possibly needed) to protect the oxidized selenocompound from thermal deselenation. 

Based on the conservation of cysteines in deiodinases and similarities with other thioredoxin-fold peroxidase enzymes, the proximal (Cys124 in DIO1) and distal (Cys194 in DIO1) cysteines caught the attention of researchers in the field [11,21,22]. We showed here that Cys124 forms a selenenyl-sulfide with Sec126, which we identified as a disulfide in DIO1^U126C^ expressed in insect cells. We further showed that Cys124 is required for reduction by 1 mM GSH coupled to GSHR and NADPH. The K_M_ for GSH of the DIO1^U126C^ is 0.9 ± 0.2 mM. Our findings are in agreement with those of Croteau et al., who reported impaired activity of DIO1^C124A^ at 5 mM GSH, but unaffected activity with DTT as reductant [11]. They also showed that the mutant was impaired in its interaction with TXN [11], while we did not find activity of the DIO1^U126C^ enzyme with TXN. Thus, it appears as if the Cys124-Cys126 disulfide is less reactive towards TXN than the authentic selenenyl-sulfide. This matter requires further investigation in the future. Using DTT in deiodinase assays, in our eyes, simply obfuscated the role of Cys124 in the mechanism by short-circuiting the deiodinase, making the endogenous Cys124 dispensable. The results presented here for human DIO1 are supported by our previous results on murine DIO3, where we showed that the proximal Cys168 is required for recycling of the enzyme after oxidation [8]. The molecular dynamics calculations of Bayse et al [15] support an attack of the proximal Cys168 (DIO3) to form a selenenyl-sulfide with the active site Sec170. A naphthalene-based small-molecule DIO3 mimetic with two chalcogens (Se/Se, Se/S, S/S) was shown to perform 5-deiodination of T4, suggesting that a proximal Cys could also directly participate and enhance the deiodination reaction [23].

We are still lacking a full understanding of the role of the distal Cys (Cys194 in DIO1, Cys209 in DIO2, and Cys239 in DIO3). A potential role is the formation of a Sec126-Cys194 selenenyl-sulfide or a Cys124-Cys194 disulfide similar to the disulfide suggested in DIO3 [8]. We tried to identify a disulfide of the active site Cys126 (or Cys124 in the same peptide) with the distal Cys194 in DIO1^U126C^, but we were not able to find any positive evidence for it in our mass spectrometry data. Such an intermediary would be appealing based on similar disulfides in other thioredoxin-fold peroxidases [24]. However, our kinetic data presented here using DIO1^U126C^ mutants with a C194A substitution did not reveal a role for Cys194 in the catalytic cycle. In contrast, it is generally assumed that the DIO1 inhibitor propyl-thiouracil (PTU) reacts only with a selenenyl-sulfide species, not with free Sec. Since PTU was able to inhibit rat DIO1^C124A^ [22], it could be possible that it attacked a Sec126-Cys194 species. Our attempts, using mass spectrometry, to find evidence for a PTU-adduct of DIO1 were, however, unsuccessful. Thus, the role of the distal Cys remains elusive.

### 3.2. The Mechanism: Proton Relay to the Active Center?

The alignment in Figure 1B shows that from Asp148 (in DIO1), all DIO isoenzymes share a stretch of invariant amino acids that was recognized early on as being close to the active site [21]. This proposition is supported by the crystal structure of murine DIO3 [8]. Mutations to amino acids in this region almost invariably inactivated the enzymes [21], but the exact roles of the involved amino acids remain unclear. Based on the structure of DIO3 [8], we have proposed a hydrogen-bonded network that would provide the proton needed by the substrate after abstraction of I^+^ (Figure 7A). Our work on DIO1 presented here supports this idea. 

In contrast, Bayse et al. suggested that Glu200 (Glu156 in DIO1) plus Asp211 and Trp207 are part of an omega loop that can create a substrate-binding site in DIO3 [15]. In their eyes, proton transfer is not the primary role of these amino acids. While the truth may lie in the middle, we would like to point out that the mutation of His174 to Ala may speak in favor of proton transfer. His174 was found very early to be essential in rat DIO1 [25]. Chemical derivatization with DEPC or photooxidation with rose-bengal, both considered His-specific reagents, inactivated the enzyme. We showed here that an H174Q mutant, which should be sterically similar, lost all its activity. In contrast, an H174A mutant, which creates a void in place of an aromatic imidazole ring, is active. Since His174 is exposed to the solvent, it is easy to imagine that removal of the ring allows Glu156 direct access to solvent and proton transfer, while a Gln174 would block access to water and only hydrogen-bond with Glu156 without proton transfer. Why rat DIO1, in contrast to human DIO1, tolerates an H174Q mutation [25], remains to be determined.

### 3.3. The Dimer

Deiodinases are only active as dimers [26,27]. While our initial data supported the idea of a disulfide-bridged DIO1 homodimer, we came to the conclusion that our present evidence is not convincing. Speaking in favor of the disulfide-bonded dimer is the observation of a band in the SDS-PAGE at mobility corresponding to a dimer, which is sensitive to reducing chemicals, and contains DIO1-derived peptides. In addition, mutations C95A and C105S abolished the “dimer” band in Western blot experiments. However, we failed to obtain evidence for intermolecular disulfides (Cys95-Cys95, Cys95-Cys105, or Cys105-Cys105) of DIO1 by mass spectrometry. Moreover, in COS7 cells, we did not see the “dimer” band in SDS-PAGE followed by Western blotting. Finally, an attempt to create a dimer in the DIO2^S84C^ variant was unsuccessful. Aggregating all this evidence, we assume that the “dimer” is a result of the massive overexpression of DIO1^U126C^ in insect cells, and does not represent the native form of DIO1. The C95A and C105S mutations moderately reduced recombinant overexpression in insect cells and this may have been sufficient to prevent formation of the “dimer”.

## 4. Materials and Methods

### 4.1. Cloning and Site-Directed Mutagenesis

Site-directed mutagenesis was performed on C-terminal Flag-tagged human DIO1 cloned into pcDNA3 [28] using the Quick Change Lightning kit (Agilent, Waldbronn, Germany). Primers used for site-directed mutagenesis are given in Table S1 (see section for Supplementary Materials). Mutated DIO1 constructs were subcloned into the pUltraBac1 donor plasmid using the primers *DIO1 fwd* and *DIO1 rev*. The *DIO1* reverse primer introduced an enterokinase cleavage sequence and an 8xHis-tag at the C-terminal end of the protein.

### 4.2. Cell Culture

*Spodoptera frugiperda* (Sf9) cells were cultivated using serum-free Sf-900™ III SFM growth medium (Gibco, Waltham, MA, USA) without additional supplements. *Trichoplusia* niBTI-Tn-5B1-4 (High Five, Hi5) cells were cultured in Express Five™ SFM growth medium (Gibco) supplemented with 20 mM Glutamax (Gibco) and 10 mg/L gentamicin (Thermo Fisher Scientific, Waltham, MA, USA). Both insect cell lines were cultured at 28 °C and without CO_2_ supplementation.

Sf9 cells at passage numbers below 20 were used for bacmid transfections, baculovirus production, and baculovirus expansion. Hi5 cell cultures were used exclusively for protein expression and activity assays.

COS7 cells (*Cercopithecus aethiops*) were cultured in DMEM/F12 (1:1) (Gibco) + 10% fetal calf serum (FCS; Gibco) + 1% penicillin (5000 U/mL)/streptomycin (5000 µg/mL) (Gibco) in a humidified atmosphere at 37 °C and 5% CO_2_. COS7 cells were used for stable transfection of DIO1 constructs.

### 4.3. Bacmid Formation in Sf9 Cells

pUltraBac1 constructs carrying sequences of different DIO1 mutants were used for the production of *Autographa californica* multiple nucleopolyhedrovirus-derived bacmids by the transformation of *E. coli* DH10Bac cells following the Bac-to-Bac^®^ TOPO^®^ protocol (Thermo Fisher Scientific) [29].

Cellfectin^®^ II (Thermo Fisher Scientific) was used to transduce Sf9 cells with isolated and purified bacmids according to the manufacturer’s protocol. The baculovirus containing supernatant (P1 stock) was collected 96 h after transfection. For generation of a second viral stock (P2) 2 × 10^7^ Sf9 cells were seeded in a T175 flask (Sarstedt, Nümbrecht, Germany) filled with 25 mL medium and inoculated with 1.5 mL of the P1 stock. The P2 viral stock was collected after 96 h. Successful transduction of Sf9 cells and the generation and amplification of the baculovirus were checked by the co-expression of an enhanced green fluorescent protein (eGFP) gene, also encoded in the pUltraBac1 donor plasmid. Viral titration was carried out following the assay published by Philipps et al [30].

### 4.4. Transfection of Hi5 Cells for Recombinant Protein Expression of DIO1 Variants and Protein Preparation

Hi5 cells were grown in T175 flasks until they reached 90–100% confluence. Cells were gently scraped, counted and seeded at a cell density of 1 × 10^6^ cells/mL into a 250 mL Erlenmeyer flask containing 50 mL medium. Suspension cultures were incubated in the dark in a shaking incubator at 28 °C and 120 rpm.

At a cell density of 2 × 10^6^ cells/mL, Hi5 suspension cultures were transduced with a DIO1-expressing baculovirus P2 stock and an optimized multiplicity of infection (MOI) of 1 for 48 h, at 120 rpm and 28 °C in the dark.

Cell pellets of DIO1-expressing Hi5 cells were then collected by centrifugation (5 min, 1000× *g*, 4 °C). Pellets from DIO1 stably expressing COS7 cells were harvested from T175 flasks. The pellets were next lysed by short sonication (six to eight 2 s pulses at 200 W) and then resuspended in homogenization buffer (250 mM sucrose; 20 mM HEPES; 1 mM EDTA in distilled H_2_O, pH 7.4). Centrifugation (1 h, 25,000× *g*, 4 °C) of lysed protein enriched DIO1 in the membrane fraction. Pellets obtained after centrifugation were again resuspended in homogenization buffer and used for quantification.

### 4.5. Transfection of COS7 Cells

Transfection of COS7 cells with wild-type and mutated DIO1-pcDNA3 constructs was performed as previously described [8].

### 4.6. Western Blotting

Membrane fractions from Hi5 and COS7 cells, respectively, were separated on 10% SDS-polyacrylamide gels containing sodium dodecyl sulfate and either stained with Coomassie Brilliant Blue R-250 (Carl Roth, Karlsruhe, Germany) or transferred on nitrocellulose membranes. Membranes were probed with antibodies against 6xHis-tag (1:1000, RRID:AB_444306), Flag-Tag (1:1000, RRID:AB_259529), and ß-Actin (1:30,000, RRID:AB_262011) diluted in 5% skimmed milk powder in PBS + 0.3% Tween. Secondary anti-mouse antibody (RRID:AB_10015289) was diluted 1:15000.

### 4.7. Radioenzymatic Assay for 5′-Deiodination

Radioactive ^125^I-labeled rT_3_ was purchased from Hartmann Analytic (Brunswick, Germany), purified as described in Kinne et al. [31], and diluted in activity assay buffer (200 mM potassium phosphate buffer (pH 6.8) plus 2 mM EDTA, 328 µM NaOH, 2 µM rT_3_ (Sigma-Aldrich, St. Louis, MO, USA)) plus regenerating reductants. A total of 20 mM DTT (Applichem, Darmstadt, Germany) has been used as an artificial reductant for the regeneration of DIO1 enzymatic activity. TXN1, GRX1, GSH, and GR have been used as described in Schweizer et al. [8].

Samples were measured in triplicates using 100–125 µg membrane protein diluted in 50 µL homogenization buffer. Protein samples were incubated with 50 µL activity assay buffer for 1 h at 37 °C. The reaction was stopped by adding stopping solution (10% bovine serum albumin, 10 mM propylthiouracil (PTU)). Proteins were precipitated using 10% ice-cold trichloroacetic acid followed by centrifugation (10,000 rpm, 5 min, 4 °C). The supernatant that contained released ^125^I^−^ was transferred to Dowex^®^50WX2 (Merck KGaA, Darmstadt, Germany) columns and eluted with 10% acetic acid. The specific enzymatic activity of each DIO1 construct was expressed as pmol of released ^125^I^−^ per mg protein per minute. 

For determination of K_M_, serial dilutions (0.5–10 mM) of GSH were prepared and incubated with the protein samples for 1 h at 37 °C as described above.

### 4.8. Detection of Disulfide Bonds

In total, 3000 µg membrane protein diluted in 400 µL homogenization buffer obtained from Hi5 cells was incubated for 3 h at 37 °C on a benchtop shaker, in a 1:1 ratio with activity assay buffer containing 1 µM rT_3_ and without any reductants and co-substrates. In addition, DIO1^U126C^ samples were incubated in activity assay buffer containing 2 mM PTU. In total, 120 µL of 200 mM N-Ethylmaleimide (NEM, Sigma-Aldrich), a specific thiol blocking agent [32], was added to each sample and incubated at room temperature for 2 additional hours. For some SDS-PAGE experiments, samples of each mutant were incubated with two different reductants: 20 mM DTT for 1 h at 37 °C, or 20 mM TCEP (Sigma-Aldrich) for 5 min at room temperature [33], and were loaded together with non-reduced samples onto non-reducing SDS-polyacrylamide gels.

### 4.9. Mass-Spectrometry-Based Analysis

Gel slices collected from the Coomassie-stained SDS-polyacrylamide gels were subjected to in-gel digestion [34,35]. Slices were washed consecutively with water, 50% acetonitrile (ACN, Sigma-Aldrich), and 100% ACN. Dried slices were next incubated with 330 ng sequencing grade chymotrypsin or trypsin (Promega, Madison, USA) at 37 °C overnight. The peptide extracts were separated and the remaining peptides extracted with 50% ACN. 

For the LC-MS analysis, dried peptides were dissolved in 10 µL 0.1% formic acid. A total of 2 µL was injected onto a C18 analytical column (self-packed 350 mm length, 75 µm inner diameter, 1.9 μm particles, ReproSil-Pur 120 C18-AQ, 1.9 µm, Dr. Maisch GmbH, Ammerbuch, Germany). Peptides were separated during a linear gradient from 2% to 35% of 90% ACN and 0.1% formic acid within 30 min at a flow rate of 300 nl/min. The nano-HPLC system was coupled online to an Orbitrap Velos mass spectrometer (Thermo Fisher Scientific). Peptide ions between *m*/*z* = 330 and *m*/*z* = 1600 were scanned in the Orbitrap detector with a resolution of 30,000 (maximum fill time 100 ms, AGC target 200,000). Top 20 precursor ions (threshold 5000, isolation width 1.2 Da), were subjected to collision-induced dissociation, and fragments were analyzed in the ion trap detector. Fragmented peptide ions were excluded from repeat analysis for 10 s.

Raw data processing and analysis of database searches were performed with Proteome Discoverer software 2.5.0.400 (Thermo Fisher Scientific). Peptide identification was performed with an in-house Mascot server version 2.6.1 (Matrix Science Ltd, London, UK) from Proteome Discoverer. MS2 data were searched against a database of common contaminants (cRAP of the Global Proteome Machine), DIO1 sequences, and appropriate background proteomes (Trembl sequences of the Plusiinae subfamily for Hi5 samples). Precursor ion *m/z* tolerance was 10 ppm, fragment ion tolerance 0.5 Da. Peptides with up to two missed cleavages were searched. The following dynamic modifications were searched: oxidation of methionine, modification of cysteine by mono-/di-/trioxidation, dehydro (for disulfide bonding), N-ethylmaleimide, oxidation+N-ethylmaleimide, and diiodothyronine (3,5-T_2_) with or without hydrogen loss (C_15_H_12/13_I_2_NO_4_, monoisotopic mass shift 523.88558/524.89340 Da). Mascot results from searches were evaluated by a fixed-value procedure and manual inspection. Spectra without high confident matches were sent to a second-round Mascot search with semi (chymo)tryptic specificity.

For the disulfide bond prediction, the DiANNA software (Boston College, Chestnut Hill, MA, USA) was employed [36]. The Xcalibur software (Thermo Fisher Scientific) was used for the search and analysis of the inter- and intramolecular disulfide bond formation, whereas the Skyline software (MacCoss Lab, University of Washington, Seattle, WA, USA) was utilized in the comparison of MS1-spectra [37].

The mass spectrometry proteomics data have been deposited in the ProteomeXchange Consortium via the PRIDE partner repository with the dataset identifier PXD032784 and 10.6019/PXD032784.

## 5. Conclusions

This work presents the first direct evidence that DIO1 forms an intramolecular selenenyl-sulfide with Cys124. The proximal Cys124 is required for reduction by GSH at physiological concentration. Thus, GSH is likely a physiological reducing substrate (co-factor) for DIO1 and the selenenyl-sulfide is a likely intermediate in the DIO1 reaction cycle.

## Data Availability

The mass spectrometry proteomics data have been deposited in the ProteomeXchange Consortium via the PRIDE (https://www.ebi.ac.uk/pride/archive/, accessed on 1 January 2020) partner repository with the dataset identifier PXD032784 and 10.6019/PXD032784.

## Supplementary Materials

The following supporting information can be downloaded at: https://www.mdpi.com/article/10.3390/ijms23105361/s1.
