# Supplementary material for: Insights into the Mechanism of Human Deiodinase 1
Source: Int J Mol Sci. 2022 May 11;23(10):5361. doi: 10.3390/ijms23105361 (PMC9141512; doi:10.3390/ijms23105361)
Supplement: Supplementary file 1 [file ijms-23-05361-s001.zip › ijms-1695463-supplementary.pdf]

Table S1. Primers used for cloning and site-directed mutagenesis.

Mutation		Sequence (5′ - 3′)
**DIO1**	fwd	ATCAGAATTCATGGGGCTGCCCCAGCCAGGG
	rev	TAAAAGCTTTTAGTGATGATGATGATGATGATGATGCTTGTCGTCGTCGTCAC TGTGGAGCTTTTCCAGAAC
**C95A**	fwd	GTCTGGCCCCAAACGCCCCGGTGGTCCGC
	rev	GCGGACCACCGGGGCGTTTGGGGCCAGAC
**C105S**	fwd	CCTCTCAGGACAGAGGTCCAACATTTGGGAGTTTA
	rev	TAAACTCCCAAATGTTGGACCTCTGTCCTGAGAGG
**S123A**	fwd	CACTGGTGCTGAATTTTGGAGCTTGTACCTGACCTTCAT
	rev	ATGAAGGTCAGGTACAAGCTCCAAAATTCAGCACCAGTG
**C124A/U126A**	fwd	CTGGTGCTGAATTTTGGAAGTGCTACCGCACCTTCATTTATGT
	rev	ACATAAATGAAGGTGCGGTAGCACTTCCAAAATTCAGCACCAG
**C124A/U126C**	fwd	CTGGTGCTGAATTTTGGAAGTGCTACCTGCCCTTCATTTATGT
	rev	ACATAAATGAAGGGCAGGTAGCACTTCCAAAATTCAGCACCAG
**T125A**	fwd	ACTGGTGCTGAATTTTGGAAGTTGTGCCTGACCTTCATT
	rev	AATGAAGGTCAGGCACAACTTCCAAAATTCAGCACCAGT
**T125S**	fwd	ACTGGTGCTGAATTTTGGAAGTTGTAGCTGACCTTCAT
	rev	ATGAAGGTCAGCTACAACTTCCAAAATTCAGCACCAGT
**U126A**	fwd	GGTGCTGAATTTTGGAAGTTGTACCGCACCTTCATTTATGTTCAAATTTGAC
	rev	GTCAAATTTGAACATAAATGAAGGTGCGGTACAACTTCCAAAATTCAGCACC
**U126C**	fwd	CTGAATTTTGGAAGTTGTACCTGCCCTTCATTTATGTTCAAATTTGA
	rev	TCAAATTTGAACATAAATGAAGGGCAGGTACAACTTCCAAAATTCAG
**U126S**	fwd	GGTGCTGAATTTTGGAAGTTGTACCTCACCTTCATTTATGTT
	rev	AACATAAATGAAGGTGAGGTACAACTTCCAAAATTCAGCACC
**Y153F**	fwd	AGCAGATTTTCTTGTCATTTTCATTGAAGAAGCACATG
	rev	CATGTGCTTCTTCAATGAAAATGACAAGAAAATCTGCT
**E156T**	fwd	CTTGTCATTTACATTGAAACAGCACATGCATCAGATG
	rev	CATCTGATGCATGTGCTGTTTCAATGTAAATGACAAG
**H174A**	fwd	CAACATGGACATCAGAAATGCCCAGAACCTTCAGGATC
	rev	GATCCTGAAGGTTCTGGGCATTTCTGATGTCCATGTTG
**H174Q**	fwd	CAACATGGACATCAGAAATCAGCAGAACCTTCAGGATC
	rev	GATCCTGAAGGTTCTGCTGATTTCTGATGTCCATGTTG
**C194A**	fwd	CAGGAGCCCCCAGGCCCCTGTGGTGGTG
	rev	CACCACCACAGGGGCCTGGGGGCTCCTG
